# Progress in Surgery

**Published:** 1897-01-02

**Authors:** 


### Progress in Surgery.

### DISEASES OF THE UPPER AIR PASSAGES.

**Pseudo-membranous Rhinitis.**—The conviction that pseudo-membranous rhinitis is in reality diphtheria in a very large proportion of cases is becoming so general amongst those who have paid any attention to the bacteriological examination of these cases, that probably few rhinologists have failed to grasp the clinical importance of treating such cases as possible sources of contagion. In bringing this subject before the Belgian Society of Otology and Laryngology, Eeman[1] stated that he had studied eleven cases; all were primary nasal diphtheria. Eight cases were pure diphtheria (long and short forms); in two there were also streptococci and staphylococci; in one bacilli and diplococci. The age of the patients varied between seventeen months and eleven years. Post-operative fibrinous rhinitis is a secondary rhinitis of a completely different nature, but even this is not always so simple as some suppose, for one of the author's polyp us cases became complicated by a pure diphtheritic rhinitis. Allowing a few weeks to elapse after the final disappearance of the membrane, he removed the polyp, and eight days later a widespread nasal diphtheria reappeared.

The disease remains local, but the germs are none the less virulent, as experiments on animals prove. The patients, therefore, able to go about their ordinary duties are to be regarded as active agents in the spreading of diphtheria; and as such they must be treated not only till all trace of membrane has disappeared from the nose, but till repeated bacteriological investigations give constant negative results. Isolation and disinfection of all nasal discharges are the only methods to adopt. Bayer,[2] commenting on Eeman's remarks, thought that there were certainly cases of non-diphtheritic membranous rhinitis; the diphtheritic cases, however, were the more numerous, but they accompanied or followed diphtheria of the pharynx, larynx, &c. As for prophylaxis, he thought the child should be isolated from six to eight weeks. Bayer, who had formerly seen many cases of post-operative membranous rhinitis, had not had a single case since he commenced to use antiseptics.

**Nasal Stenosis.**—An analysis of 66 cases of turbinotomy has been made by Abercrombie,[3] all the cases having been operated on by Carmalt Jones by his "spokeshave"; only 4 cases are stated not to have benefited by the operation in greater or less degree. Of the 62 cases benefited, 21 were relieved in marked degree, decided improvement followed in 14, and in 27 the relief was only slight. The large majority of cases were young adults. In practically all cases the nasal stenosis was the result of hypertrophic rhinitis to a greater or less extent; but, in addition, there were, in 8, septal spurs; in 3, a deviated septum; in 10, nasal polypi; in 11, adenoids; in 14, otitis media (dry or purulent); in 17 cases the operation was unilateral; in the remaining 49, bilateral. Cocaine was practically useless as an anæsthetic, but operation under nitrous oxide was painless. In 30 cases no anæsthetic was used, and the pain was described as great, though momentary. The bleeding, in every case, without exception, was pretty profuse, but in no case was there alarming hæmorrhage at the time of operation. More or less bleeding occurred from the nose for several days afterwards in most of the cases. In one man secondary hæmorrhage occurred fourteen days after the operation. Most of the patients operated on returned to their homes on the day of the operation, some of them on foot. In only one case (a very anæmic girl) did the patient actually faint after the operation, though many felt "faintish" after it. A good many of the patients stayed in bed for a few days afterwards, or at any rate within doors. But not a few were not confined to bed or even the house at all. Several returned to work the following day. After-headache, more or less severe, and chiefly frontal, was present in most of the cases. In very few instances indeed were there any unfavourable symptoms following the operation, and in those few nothing alarming occurred. Slight swelling about the nose and eyes was not very uncommon for a few days. In no case, judging from patients' letters, has atrophic rhinitis resulted from the operation. As regards after-treatment, in all cases cotton-wool plugs were packed up the nostrils to arrest hæmorrhage. An antiseptic lotion, to which tannic acid was sometimes added, was prescribed for most of the patients. Some were ordered unguentum acidi borici to apply up the nostrils; and for the headache so commonly following the operation, and often complained of so bitterly, potassium bromide was usually prescribed. As a rule, for a week or so before operating, the patient was directed to use an alkaline and antiseptic nasal lotion, as a preparatory cleansing agent. In two cases, some time after operation, and presumably resulting from it, a condition of pharyngitis sicca was observed. But in both of these cases the patients admitted that even with that they were more comfortable than before the operation with their noses blocked up. Wingrave, in his microscopical examination of over 200 turbinals removed by the spokeshave, came across a few in which there were distinct evidences of atrophic changes present, so that it is possible the two cases referred to above may have been cases of atrophic rhinitis in an early stage, and not cases where the operation of turbinotomy led to atrophic mischief. In no case examined after the operation has any trouble arisen from the opening up of the hiatus maxillaris, which latter, no doubt, may happen in some cases. No external nasal deformity has been seen to result from the operation in a single case; rather the reverse, indeed, occurs. The nostrils, being stimulated by the passage of air through them, become active and patent, with the result that the nose becomes larger, and instead of being a small, deformed, and more or less useless organ, it becomes of a good size and shape, and has its function restored.

After turbinotomy a reproduction to a greater or less extent of the inferior turbinal mucous membrane may take place, and this was observed in several of the cases. More especially so was this in one patient, where a small piece of the new turbinal, so to speak, was removed and microscopically examined by Wingrave, who found it to consist of perfectly healthy and normal mucous membrane "complete in its details," and with no appearances either of atrophy or hypertrophy. In several cases ear symptoms, and especially tinnitus, were relieved.

[1] Journ. of Laryng., Oct., 1896. [2] Ibid. [3] Ibid.

